# Association of maternal serum magnesium with pre-eclampsia in African pregnant women: a systematic review and meta-analysis

**DOI:** 10.1093/inthealth/ihad026

**Published:** 2023-04-07

**Authors:** Endalamaw Tesfa, Abaineh Munshea, Endalkachew Nibret, Solomon Tebeje Gizaw

**Affiliations:** Department of Medical Biochemistry, College of Medicine and Health Sciences, Bahir Dar University, Bahir Dar, Ethiopia; Health Biotechnology Division, Institute of Biotechnology, Bahir Dar University, Bahir Dar, Ethiopia; Health Biotechnology Division, Institute of Biotechnology, Bahir Dar University, Bahir Dar, Ethiopia; Department of Biology, College of Science, Bahir Dar University, Bahir Dar, Ethiopia; Health Biotechnology Division, Institute of Biotechnology, Bahir Dar University, Bahir Dar, Ethiopia; Department of Biology, College of Science, Bahir Dar University, Bahir Dar, Ethiopia; Department of Medical Biochemistry, College of Health Science, Addis Ababa University, Addis Ababa, Ethiopia

**Keywords:** association, pre-eclampsia, review, serum magnesium

## Abstract

Pre-eclampsia (PE) is a pregnancy-related disorder characterized by hypertension and proteinuria occurring after 20 weeks of gestation. Several studies have been performed to determine the serum magnesium (Mg) level in PE, but most report inconclusive results. Consequently, this study was designed to resolve this controversy among African women. PubMed, Hinari, Google Scholar and African Journals Online electronic databases were searched for studies published in English. The qualities of included articles were appraised using the Newcastle–Ottawa quality assessment tool. Stata 14 software was utilized for analysis and serum Mg levels in cases and normotensive controls were compared through mean and standardized mean difference (SMD) at the 95% confidence interval (CI). In this review, we found that the mean serum Mg level was significantly reduced in cases (0.910±0.762 mmol/L) vs controls (1.167±1.060 mmol/L). The pooled SMD of serum Mg was significantly lower in cases (−1.20 [95% CI −1.64 to −0.75]). Therefore, since serum Mg is reduced in cases vs controls, we propose that Mg is involved in the pathophysiology of PE. Nevertheless, to know the exact mechanisms of Mg in PE development will require large-scale prospective studies.

## Introduction

Magnesium (Mg) is the fourth most abundant cation, after calcium, potassium and sodium, and it is the second most prevalent intracellular cation after potassium in human tissues.^1,2^ The human body contains approximately 21–28 g of magnesium. About 50–60% of the total Mg is stored in the bones in association with calcium and phosphate.^3–5^ Another 40–50% of the total Mg is in the intracellular fluid and only 1% of Mg is found in the extracellular environment.^3,4^ In serum, Mg exists in three forms. The free ionized form is biologically active and accounts for 60%, the second form is protein bounded and accounts for 30% and the remaining 10% is found in complexes with anions such as phosphate and citrate.^4,5^

Mg is an essential mineral naturally found in many food stuffs and serves as a cofactor for >300 enzymatic reactions in energy production, protein synthesis, nucleic acid synthesis, mitochondrial functions, neuromuscular activity, bone formation, blood pressure (BP) regulation, heart rhythm regulation, genome integrity and immune system competence.^2,6^ There are several reports that revealed the association between serum Mg and several chronic diseases, including diabetes mellitus, hypertension, pre-eclampsia (PE), hyperlipidaemia, cardiac arrhythmia and bronchial asthma, and Mg is used for the treatment of these diseases.^7^ However, a recent randomized clinical trial showed that oral Mg supplementation did not reduce the risk of PE.^8^

PE is a pregnancy-related disorder of hypertension and proteinuria occurring after 20 weeks of pregnancy.^9^ Despite the remarkable progress in the understanding of the pathophysiology of PE, the exact cause of PE is not fully understood, but evidence from animal and human studies so far have shown that failure of spiral artery remodelling causes placental ischaemia, leading to maternal syndromes.^10^ Several factors increase the risk of PE, including maternal micronutrient deficiency^11^ and maternal genetic susceptibility,^12^ and foetal factors also play a significant role in the pathogenesis of PE.^13^

The molecular mechanism of how Mg deficiency causes PE is not fully understood, but it is believed that Mg ion decreases BP by acting as a calcium antagonist, leading to reduced peripheral vascular resistance.^14^ Likewise, Mg deficiency causes a decrease in the production of nitric oxide and endothelial prostaglandin I_2_, which results in vasoconstriction and high BP.^15,16^ There are readily available tests to determine the serum Mg status, although the serum Mg level has little correlation with the total body Mg level or with Mg in specific tissues.^4,5^ Many studies have tried to determine the serum Mg level in pre-eclamptic and normotensive pregnant women, but most of these have reported controversial results. Most studies have shown a lower serum Mg level in pre-eclampsia than in normotensive pregnant women.^17–20^ In contrast, higher serum Mg levels in pre-eclampsia than in normotensive pregnant women have been reported,^21^ while some studies showed a non-significant difference in serum Mg concentrations in the two groups.^22–25^ Therefore, this review is intended to resolve this discrepancy in African pre-eclamptic and normotensive pregnant women.

## Methods

### Protocol registration

The protocol of the current study is recorded at the National Institute for Health Research (PROSPERO registration CRD42020192856).

### Study design and search strategy

A systematic review was performed on available articles to compare maternal serum Mg levels in pre-eclamptic and normotensive pregnant African women. We searched the following databases: PubMed, Hinari, African Journals Online (AJOL) and Google Scholar. The search was done by using the following Medical Subject Heading (MeSH) terms: ‘Electrolytes OR Magnesium (Mg) AND pre-eclampsia AND Africa’ separately or in combination. All published and unpublished articles through 30 September 2021 were retrieved and assessed for their eligibility. The Preferred Reporting Items for Systematic Reviews and Meta-Analyses (PRISMA) guideline was utilized to conduct this systematic review and meta-analysis.

### Eligibility criteria

Inclusion criteria were case–control and cross-sectional studies performed in pregnant African women, published and unpublished studies in English reporting serum Mg in mean±standard deviation (SD) and community and health-institution–based studies. Studies presented in conferences, editorials, reviews and clinical trials were excluded.

### Study selection and screening

**Figure 1.**
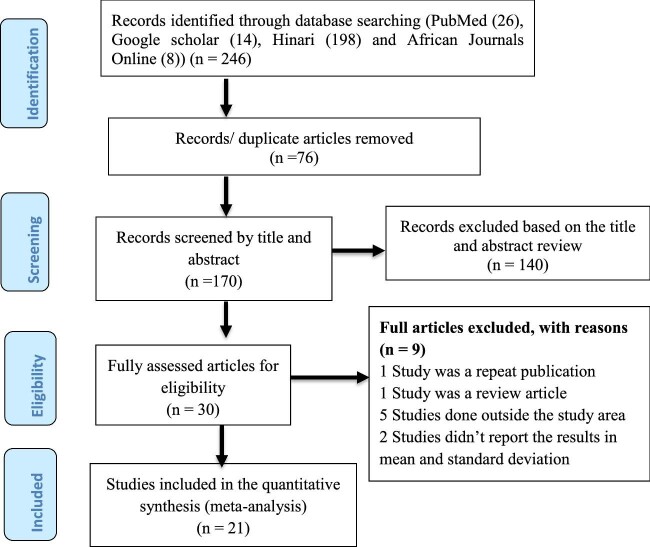
PRISMA flow chart showing studies conducted in African pregnant women.

All documents recognized by our search were transferred into EndNote X9 (Clarivate, Philadelphia, PA, USA) and identical studies were excluded. The titles and abstracts of recognized studies were evaluated by two assessors and eligible articles were included for additional review. All recorded studies were carefully reviewed before data abstraction to ensure their appropriateness. When discrepancies occurred, a third evaluator was consulted. Our search strategy is presented in a PRISMA flow chart (Figure 1).^26^

### Outcome of interest and terms

The primary objective of the current study was to evaluate the association between maternal serum Mg levels in pre-eclamptic and normotensive African pregnant women. Hypertension was defined as measured BP ≥140/90 mmHg a minimum of two times at 4-h intervals. Gestational hypertension was defined as measured BP ≥140/90 mmHg after 20 weeks of pregnancy. Pre-eclampsia was defined as measured BP ≥140/90 mmHg after 20 weeks of pregnancy plus proteinuria or the presence of severe features of the disease. Proteinuria was defined as urinary protein excretion ≥300 mg/24-h urine sample or ≥1*+* on a presumptive dipstick investigation or a total protein:creatinine ratio ≥30 mg/mmol. Eclampsia was defined as seizures in the mother with high BP that could not have another cause.^27^

### Quality assessment

The quality of the case–control studies was appraised using the Newcastle–Ottawa Scale (NOS). A modified form of the NOS quality assessment tool was utilized to appraise the quality of cross-sectional studies.^28^ In the NOS, there are three criteria to a maximum score of nine. The quality of each article was evaluated by the following procedure: a score ≥7 was ‘good’, 4–6 was ‘fair’ and ≤3 was ‘poor’. To increase the strength of our study, we included articles with good and fair quality scores.^28^

### Data extraction process

The data for this review were extracted using the Joanna Briggs Institute Reviewers’ Manual data collection instrument.^29^ The abstracts and full texts were studied by two different evaluators. Data abstraction consisted of the name of the author, year of publication, state, study strategy, number of participants, maternal age, weeks of pregnancy, BMI, laboratory determination methods, systolic and diastolic BP and mean serum Mg level. Studies reporting the mean serum Mg level in mg/dl were converted into mmol/L by multiplying by 0.4114.

### Data analysis

The data were entered into Excel (Microsoft, Redmond, WA, USA) and the meta-analysis was performed using Stata 14 software (StataCorp, College Station, TX, USA) and the paired sample t-test was performed using SPSS version 20 software (IBM, Armonk, NY, USA). A forest plot of the standardized mean difference (SMD) was used to compare the maternal serum Mg level among pre-eclamptic and normotensive pregnant women at the 95% CI. The SMD is the ratio of the mean difference to the pooled SD. The standard error of the mean (SEM) was calculated as SEM=SD/√*n*. Variables such as mean maternal age, mean gestational age, mean BMI, mean systolic BP, mean diastolic BP and mean Mg were evaluated using a paired sample t-test for their difference between cases and controls.

### Heterogeneity and publication bias

Heterogeneity was assessed using Cochran’s Q, the I^2^ statistic and p-value. An I^2^ statistic value <25%, 25–50% and ≥50% indicated little, average and high heterogeneity, respectively. In this study we utilized the random effects model (REM) for data analysis. Sensitivity tests were performed to determine the influence of independent variables and publication bias was judged using funnel plots and Egger’s test.

## Results

### Study selection

In this study, 246 studies were identified in four databases using a combination of search terms and 170 studies were found to be suitable for title and abstract evaluation after excluding 76 identical studies. A total of 140 articles were excluded by their title and abstract assessment. A total of 30 articles underwent full-text assessment for eligibility and 9 studies were excluded (5 were done outside the study area, 2 studies did not’ report the result as mean±SD and the others were repeated publications and a review article). That left a total of 21 studies that were included in this review.^17–25,30–41^

### Study characteristics

Fourteen studies were case–control^17,19,20,22–25,30,34,35,38–41^ and 7 were cross-sectional.^18,21,31–33,36,37^ Studies that were conducted in Africa and published through 30 September 2021 were included. Fifteen studies were conducted in Nigeria, three in South Africa, one in Ghana and one each in Sudan and the Democratic Republic of Congo. The current study included 2398 pregnant women (1276 cases and 1122 controls) (Table 1).

**Table 1.** Characteristics of research articles included in this review (N = 21)

	Authors &		Sample	NP	PE	Study	Laboratory Assay	PE Mg mmol/L	NP Mg mmol/L	Quality
No	Publication Year	Country	Size	(n = 1122)	(n = 1276)	Design	Methods	Mean± SD	Mean ±SD	Score
1	Adekanle et al., 2014^30^	Nigeria	150	75	75	Case-control	Photometric	0.58 ± 0.17	0.73 ± 0.14	6 Points
2	Adewolu, 2013^21^	Nigeria	40	20	20	Cross-sectional	Photometric	1.02 ± 0.4	0.85 ± 0.2	5 Points
3	Akinloye et al., 2010^31^	Nigeria	89	40	49	Cross-sectional	AAS	0.5 ± 0.2	1.0 ± 0.2	4 Points
4	Allagoa et al., 2019^25^	Nigeria	104	52	52	Case-control	Photometric	0.74 ± 0.1	0.77 ± 0.15	7 Points
5	Atiba et al., 2020^17^	Nigeria	74	37	37	Case-control	AAS	0.53 ± 0.06	0.69 ± 0.08	7 Points
6	Darkwa et al., 2017^24^	Ghana	60	30	30	Case-control	AAS	0.7 ± 0.15	0.76 ± 0.14	7 Points
7	Enebe et al., 2020^18^	Nigeria	162	81	81	Cross-sectional	AAS	0.57± 0.40	2.07 ± 2.71	7 Points
8	Idogun et al., 2007^32^	Nigeria	34	23	11	Cross-sectional	Photometric	0.62 ± 0.12	0.64 ± 0.13	5 Points
9	Igberase et al., 2007^33^	Nigeria	130	65	65	Cross-sectional	Photometric	0.69 ± 0.14	1.06 ± 0.33	4 Points
10	Khedun et al., 1998^23^	SA	81	27	54	Case-control	AAS	0.81 ± 0.03	0.84 ± 0.03	5 Points
11	Maduray et al., 2017^22^	SA	66	23	43	Case-control	ICP-OES	1.23 ± 0.02	1.59 ± 0.06	7 Points
12	Nura, et al., 2012^19^	Nigeria	192	64	128	Case-control	Photometric	0.93 ±0.36	1.050 ± 0.28	5 Points
13	Odigie et al., 2004^34^	Nigeria	68	32	36	Case-control	Photometric	0.23 ± 0.03	0.27 ± 0.03	4 Points
14	Okoror et al., 2020^20^	Nigeria	81	54	27	Case-control	Photometric	0.96 ± 0.14	0.99 ± 0.07	6 Points
15	Olusanya et al., 2015^35^	Nigeria	156	78	78	Case-control	Photometric	0.82 ± 0.05	0.86 ± 0.07	7 Points
16	Omotayo et al., 2018^36^	Nigeria	192	64	128	Cross-sectional	Photometric	0.93 ± 0.36	1.05 ± 0.28	6 Points
17	Onyegbule et al., 2014^37^	Nigeria	102	48	54	Cross-sectional	AAS	4.04 ± 0.29	5.43 ± 0.45	4 Points
18	Richards et al., 2013^38^	SA	192	96	96	Case-control	ICP-OES	0.87 ± 0.21	0.72 ± 0.07	7 Points
19	Sidahmed et al., 2017^39^	Sudan	120	60	60	Case-control	Photometric	0.53 ± 0.09	0.75 ± 0.13	5 Points
20	Ugwuja et al., 2016^40^	Nigeria	80	40	40	Case-control	AAS	1.32 ± 0.45	1.73 ± 0.32	7 Points
21	Wakumilua et al., 2018^41^	DRC	225	113	112	Case-control	Photometric	0.49 ± 0.17	0.64 ± 0.06	6 Points

AAS- Anomic absorption spectrometry, DRC- Democratic Republic of Congo, ICP-OES- Inductively coupled plasma—optical emission spectrometry PE- Pre-eclampsia, NP- Normal pregnant, Mg- Magnesium, mmol/L- Millimole per liter, N/n- sample size, SA-South Africa, SD- Standard deviation.

#### Association of independent variables with pre-eclampsia

In this review, we computed the mean values of variables in pre-eclamptic and normotensive African pregnant mothers. Non-significant association has been observed among the study groups in respect to the mean values of maternal age, gestational age, and body mass index. However, significant association has been observed in the mean values of systolic and diastolic blood pressure with serum Mg level between the cases and controls (Table 2).

**Table 2.** Paired sample t-test of variables associated with pre-eclampsia

**S. No**	**Variable**	**Studies**	**Cases (N = 1276)**	**Controls (N = 1122)**	**P-value**
1	Age in year (Mean ± SD)	17	28.22 ± 2.30	28. 48 ± 2.67	0.337
2	GA in week (Mean ± SD)	12	32.78 ± 5.81	32.61 ± 6.74	0. 838
3	BMI (Mean ± SD)	12	29.17 ± 1.94	28.26 ± 5.39	0. 491
4	SBP (Mean ± SD)	13	163.28 ± 10.20	110.11 ± 3.39	≤ 0.001**
5	DBP (Mean ± SD)	13	105.17 ± 6. 43	69.15 ± 2.41	≤ 0.001**
6	Mg (mmol/L)	21	0.910 ± 0.762	1.167 ± 1.06	0.013**

BMI- Body mass index, DBP- Diastolic blood pressure, GA- Gestational age, SBP- Systolic blood pressure, SD- Standard deviation ** statistically significant at p<0.05.

### Serum mg level in pre-eclamptic and normotensive pregnant women

**Figure 2.**
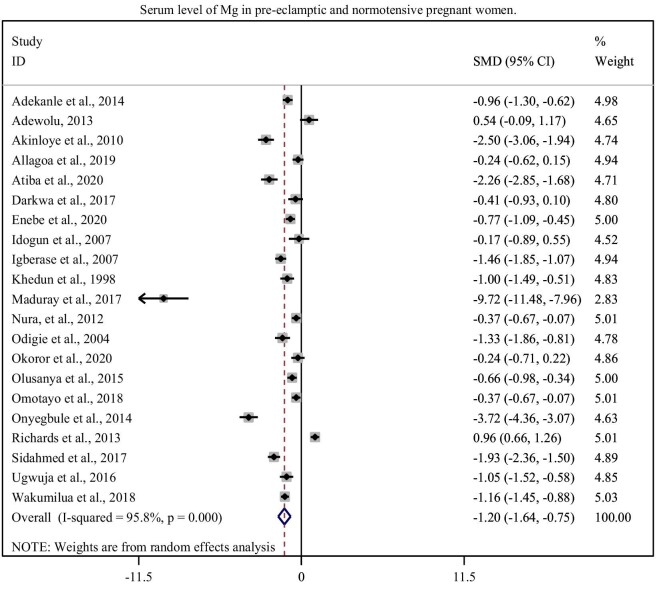
Forest plot of SMD of serum Mg in pre-eclamptic and normotensive pregnant women.

In this sub-categorical analysis, 21 observational studies were included to compare serum Mg level between pre-eclamptic and normotensive pregnant women. Fifteen of the included studies showed a significant reduction of serum Mg level in pre-eclamptic women as compared with normotensive pregnant women.^7–19,22,23,30,31,33–37,39–41^ Although, five studies^20,21,24,25,32^ showed non-significant association but one study^8^ showed a significant increment in the serum levels of Mg in cases than normotensive controls. The pooled meta-regression analysis showed that there is a statistical significant reduction in the serum Mg level among the cases than controls with the overall combined SMD of −1.20 (95% CI: −1.64, −0.75) (Figure 2).

### Sensitivity test and publication bias

**Figure 3.**
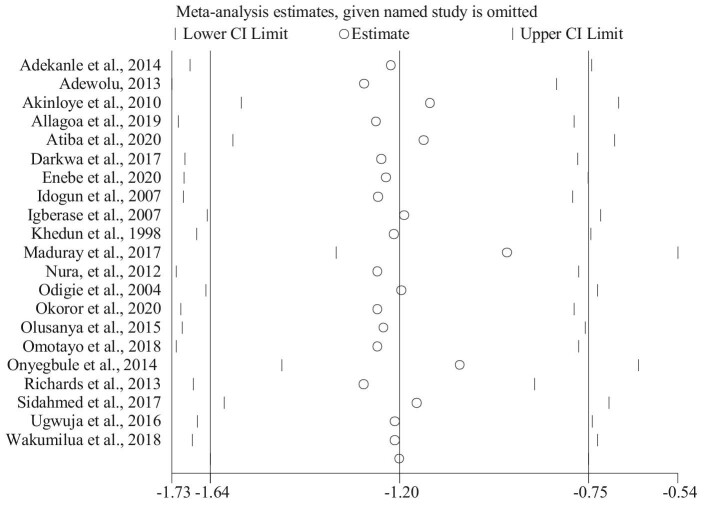
Influence analysis of serum Mg in pre-eclamptic and normotensive pregnant women.

In this review we performed sensitivity test by removing a single study at a time to know the consistency of the findings. There has no substantial variation in the combined SMD once removing one of the studies at 95% CI (Figure 3). This indicates that there is no individual study that extremely influences the combined values of serum Mg concentration among the two groups. Additionally, the funnel plot didn't show any evidence of publication bias between the serum Mg level in pre-eclamptic and normotensive pregnant women. Besides, Egger's test didn't show evidence of publication bias (p-value = 0.53).

## Discussion

This study is the first systematic review and meta-analysis in Africa which provides baseline information on the association of maternal serum Mg level in pre-eclamptic and normotensive pregnant women. In this study, maternal age, gestational age and BMI were comparable between the two groups and these variables didn't showed statistical difference between the two groups. The mean systolic and diastolic blood pressure readings were significantly higher in pre-eclamptic women as compared to those normotensive pregnant women with the p-value of <0.001, as it is defined in the case definitions of this review.

In this study, we found that the mean maternal serum Mg level was significantly reduced in pre-eclamptic women as compared with normotensive pregnant women (0.910 ± 0.762 vs 1.167 ± 1.06 mmol/L) with the overall SMD of Mg (SMD = −1.20, 95% CI: −1.64, −0.75). Similar finding were reported in the studies conducted in Nigeria and China.^18,42^ This low serum Mg level might be attributed to an inadequate dietary intake or an increased Mg loss from the body. Even though, decreased serum Mg level does not always indicate the actual nutritional status of the women, because of the low percentage of Mg in the extracellular environment.^43^ There are reports which failed to clearly show oral Mg supplementation and risk of PE.^8,44^

Mg sulfate is the preferred pharmacological intervention for the prevention and treatment of seizures in the women with severe PE, even though, the mechanism of action of Mg sulfate in eclampsia prophylaxis and treatment remains poorly understood.^45^ However, it is believed that dilation of cerebral blood vessels reduces cerebral ischemia and its action related to of Mg acting as calcium receptor blocker of muscle contraction.^46^ Mg sulfate causes peripheral vasodilation by blocking calcium receptor via inhibiting *N*-methyl-D aspartate receptors in the brain. It also alters the neuromuscular transmission via competitively blocking the entry of calcium into synaptic endings.^46^ Mg sulfate treatment showed a significant reduction in the risk of convulsion by 52% and 67% as compared to diazepam and phenytoin treatment, respectively. Mg sulfate treatment for severe pre-eclampsia reduces the risk of progression to eclampsia by more than half and also reduces maternal mortality. As well, Mg sulfate treatment showed a significant improvement in the neonatal outcomes as compared to phenytoin treatment.^43^

Mg is an essential micronutrient that is required in every cell and organism as an activator of several enzymes, bone formation, boosting immune system, mitochondrial functions, neuromuscular activity, proteins synthesis and DNA synthesis.^6,47^ In the blood and serum, Mg is mostly bound to serum albumin and stored in muscle fibers and in bones. The biologically active form is the ionized which is measured in the plasma.^47^ The serum level of Mg is maintained by the interaction between gastro-intestinal Mg absorption and renal Mg excretion.^47^ The intra-cellular Mg and bone Mg level did not play an active role in the regulation of blood Mg concentration; while a major role is played by the renal tubule, which adapts to match the urinary magnesium excretion and net entry of magnesium into extracellular fluid.^47^

The molecular mechanism of pre-eclampsia is not fully understood, but poor placentation cause's placental ischemia and maternal syndrome which resulted in hypertension and proteinuria.^48^ Maternal micronutrient deficiency and maternal genetic susceptibility play a substantial role in the etiopathogenesis of PE.^49,50^ It is believed that Mg ion decrease the blood pressure by blocking calcium channel and by reducing peripheral vascular resistance.^14^ Mg ion affects blood pressure by modulating vascular tone and structure through different biochemical reactions that control vascular contraction/dilation, growth/apoptosis, differentiation and inflammation. It also stimulates production of vasodilator prostacyclin and nitric oxide and it alters vascular responses to vasoconstrictor agents.^15,51^

The serum Mg is tightly regulated by the interaction of intestine, kidney, bone and parathyroid hormone.^5^ The measured serum Mg concentration may be increased in patients with severe acute or chronic renal failure. In this case ionized and total Mg concentrations occasionally increased in individuals with renal failure. Low serum Mg concentration may be observed in nutritional deficiency and in chronic diseases such as: hypertension, diabetes mellitus, coronary heart disease, and osteoporosis.^52^ In this review, we found that the mean serum Mg level was significantly low in pre-eclamptic women as compared with normotensive pregnant women that is might be due to nutritional deficiency.

## Strengths and Limitations

This systematic review and meta-analysis generates pooled data that compared the serum Mg level in pre-eclamptic and normotensive African pregnant women. In addition, this review serves as the baseline information for further study. The study is not free from some form of limitations specially, the search strategy was limited to articles published in English, and this could lead to reporting bias. Most of the included studies originated from one country Nigeria, and this may influence its generalizability to African pregnant women. Besides, presence of high statistical heterogeneity among the included studies would decrease the generated evidence of this review.

## Conclusions

The mean maternal serum Mg level was significantly reduced in pre-eclamptic women as compared with normotensive pregnant women. The overall pooled SMD of serum Mg level was also significantly decreased in cases than controls. Thus, we conclude that Mg could play certain roles in the etiopathogenesis of PE. However, concrete evidence on the functions of Mg and risk of PE pathogenesis in African pregnant women would require large scale prospective studies.

## Abbreviations

AAS- Anomic absorption spectrometry

BMI- body mass index

CI- confidence interval

DBP- diastolic blood pressure

DRC- Democratic Republic of Congo

GA- gestational age

ICP-OES- Inductively coupled plasma—optical emission spectrometry

Mg- magnesium,

mg/dL- milligram per deciliter

mmHg- millimeter of mercury

mmol/L- millimole per liter

N/n- sample size

NOS- Newcastle- Ottawa Scale

NP- Normal pregnant,

PE- Pre-eclampsia

PRISMA- preferred reporting items for systematic reviews and meta-analyses

SBP- systolic blood pressure

SA-South Africa

SD- standard deviation

SEM- standard error of mean

SMD- standardized mean difference

## Data Availability

All data pertaining to this study are contained and presented in this document and in the supplementary files.

## References

[1] Okwi AL , Bimenya GS, Tumwine LK et al. The reference range of serum magnesium substance concentration among healthy young adults at Makerere University College of Health Sciences 2012. Tanzan J Health Res. 2016;18(2):1–7.

[2] Schwalfenberg GK , Genuis SJ. The Importance of Magnesium in Clinical Healthcare. Hindawi Scientifica. 2017;4179326:1–15. 10.1155/2017/4179326 PMC5637834 29093983

[3] Konrad M , Schlingmann KP, Gudermann T. Insights into the molecular nature of magnesium homeostasis. Am J Physiol Renal Physiol. 2004;286:F599–605. 15001450 10.1152/ajprenal.00312.2003

[4] Lum G. Clinical Utility of Magnesium Measurement. Lab Med. 2004;35(2):106–11.

[5] Reddy ST , Soman SS, Yee J. Magnesium Balance and Measurement. Adv Chronic Kidney Dis. 2018;25(3):224–9. 29793660 10.1053/j.ackd.2018.03.002

[6] Fanni D , Gerosa C, Nurchi VM et al. The Role of Magnesium in Pregnancy and in Fetal Programming of Adult Diseases. Biol Trace Elem Res. 2021;199(10):3647–57. 33319331 10.1007/s12011-020-02513-0 PMC8360883

[7] DiNicolantonio JJ , Liu J, O'Keefe JH. Magnesium for the prevention and treatment of cardiovascular disease. Open Heart. 2018;5(e000775):1–10. 10.1136/openhrt-2018-000775 PMC6045762 30018772

[8] Araújo CAL , Oliveira LS, GusmãoIsabela MB et al. Magnesium supplementation and preeclampsia in low-income pregnant women – a randomized double-blind clinical trial. BMC Pregnancy and Childbirth. 2020;20(208):1–6. 10.1186/s12884-020-02877-0 PMC7146998 32272914

[9] Sánchez-Aranguren LC , Prada CE, Riaño-Medina CE et al. Endothelial dysfunction and preeclampsia: Role of oxidative stress. Front Physiol. 2014;5(372):1–11. 25346691 10.3389/fphys.2014.00372 PMC4193194

[10] Phipps E , Prasanna D, Brima W et al. Preeclampsia: Updates in Pathogenesis, Definitions, and Guidelines. Clin J Am Soc Nephrol. 2016;11(6):1102–13. 27094609 10.2215/CJN.12081115 PMC4891761

[11] Yakoob MY , Khan YP, Bhutta ZA. Maternal mineral and vitamin supplementation in pregnancy. Expert Rev Obstet Gynecol 2010;5(2):241–56.

[12] Mohaupt M. Molecular aspects of preeclampsia. Mol Aspects Med. 2007;28(2):169–91. 17412411 10.1016/j.mam.2007.02.005

[13] Galaviz-Hernandez C , Sosa-Macias M, Teran E et al. Paternal Determinants in Preeclampsia. Front Physiol. 2018;9(1870):1–7. 30666213 10.3389/fphys.2018.01870 PMC6330890

[14] Romani AMP. Beneficial Role of Mg^2+^ in Prevention and Treatment of Hypertension. Int J Hypertens. 2018;9013721:1–7. 10.1155/2018/9013721 PMC6016150 29992053

[15] VanWijk Mj , Kublickiene K, Boer K et al. Vascular function in preeclampsia. Cardiovasc Res. 2000;47(2000): 38–48. 10869528 10.1016/s0008-6363(00)00087-0

[16] Maier JA , Malpuech-Brugere C, Zimowska W et al. Low magnesium promotes endothelial cell dysfunction: Implications for atherosclerosis, inflammation and thrombosis. Biochim Biophys Acta. 2004;1689(1):13–21. 15158909 10.1016/j.bbadis.2004.01.002

[17] Atiba AS , Akindele RA, Bello NO et al. Serum Magnesium Levels in Second and Third Trimesters of Pregnancy in Patients That Developed Pre-Eclampsia and Feto-Maternal Outcome. Open J Obstet Gynecol. 2020;10:108–17.

[18] Enebe JT , Dim CC, Ugwu EO et al. Serum antioxidant micronutrient levels in pre-eclamptic pregnant women in Enugu, south-East Nigeria: A comparative cross-sectional analytical study. BMC Pregnancy Childbirth. 2020;20(392):1–9. 10.1186/s12884-020-03081-w PMC7339396 32631273

[19] Nura AF , Jimoh OG. Comparative study of serum Calcium, Magnesium and Uric acid in Pre-eclampsia and normal pregnancy in University of Ilorin Teaching Hospital. Unpublished. 2012:1–69.

[20] Okoror CEM , Enabudoso EJ, Okoror OT et al. Serum calcium-magnesium ratio in women with pre-eclampsia at a tertiary hospital in Nigeria. Int J Gynaecol Obstet. 2020;149:354–8. 32167585 10.1002/ijgo.13142

[21] Adewolu OF. Serum sodium, potassium, calcium and magnesium in women with pregnancy induced hypertension and preeclampsia in Oredo local Government, Benin Metropolis: A pilot study. Afr J Med Health Sci. 2013;12(1):1–5.

[22] Maduray K , Moodley J, Soobramoney C et al. Elemental analysis of serum and hair from pre-eclamptic South African women. J Trace Elem Med Biol. 2017;2017:1–7. 10.1016/j.jtemb.2017.03.004 28325649

[23] Khedun SM , Ngotho D, Moodley J et al. Plasma and red cell Magnesium levels in black African women with hypertensive disorders of pregnancy. Hypertens Pregnancy. 1998;17(2):125–34.

[24] Darkwa EO , Antwi-Boasiako C, Djagbletey R et al. Serum magnesium and calcium in preeclampsia: A comparative study at the Korle-Bu Teaching Hospital, Ghana. Dovepress. 2017;10:9–15. 10.2147/IBPC.S129106 PMC5565255 28860856

[25] Allagoa DO , Agboa OJ, Ugboma HA et al. Comparative Evaluation of Serum Magnesium Level in Pre-eclamptic and Non Pre-eclamptic Women in a tertiary Hospital Southern Nigeria. Orient J Med. 2019;31(1-2):14–22.

[26] Moher D , Liberati A, Tetzlaff J et al. Preferred reporting items for systematic reviews and meta-analyses: The PRISMA statement. PLoS Med. 2009;6(7):1–6. 10.1371/journal.pmed.1000097 PMC2707599 19621072

[27] Chaiworapongsa T , Chaemsaithong P, Yeo L et al. Pre-eclampsia part 1: Current understanding of its pathophysiology. Nat Rev Nephrol. 2014;10(8):466–80. 25003615 10.1038/nrneph.2014.102 PMC5893150

[28] Hartling L , Milne A, Hamm MP et al. Testing the Newcastle Ottawa Scale showed low reliability between individual reviewers. J Clin Epidemiol. 2013;66(9):982–93. 23683848 10.1016/j.jclinepi.2013.03.003

[29] Institute JB. Joanna Briggs Institute Reviewers’ Manual: 2014 edition /Supplement. Joanna Briggs Institute. 2014:1–40.

[30] Adekanle DA , Adeyemo OT, Adeniyi AA et al. Serum Magnesium Levels in Healthy Pregnant and Pre-Eclamptic Patients—A Cross-Section Study. Open J Obstet Gynecol. 2014;4:561–8.

[31] Akinloye O , Oyewale OJ, Oguntibeju OO. Evaluation of trace elements in pregnant women with pre-eclampsia. Afr J Biotechnol. 2010;9(32):5196–202.

[32] Idogun ES , Imarengiaye CO, Momoh SM. Extracellular Calcium and Magnesium in Pre-eclampsia and Eclampsia. Afr J Reprod Health. 2007;11(2):89–94. 20690291

[33] Igberase GO , Ebeigbe PN, Okonta PI et al. Serum Magnesium Levels in Normal and Pre-Eclamptic Gestation in Benin City. Niger Med J. 2007;48(1):21–23.

[34] Odigie IP , Anorlu RI, Adesiyun AE et al. Serum Magnesium levels in Non-pregnant, pregnant and Pre-eclamptic women in Lagos, Nigeria. Nig Qt J Hosp Med. 2004;14(2):178–80.

[35] Olusanya A , Oguntayo AO, Sambo AI. Serum levels of calcium and magnesium in pre-eclamptic-eclamptic patients in a tertiary institution. It J Gynaecol Obstet. 2015;27(3):101–10.

[36] Omotayo OL , Nura A, Olajide OAW et al. Study on Comparison of Serum Levels of Calcium, Magnesium, and Uric Acid in Mild Preeclamptics, Severe Preeclamptics, and Normal Pregnant Women in Ilorin, Nigeria. Niger J Exp Clin Biosci. 2018;3:71–77.

[37] Onyegbule O , Meludu S, Dioka C et al. Comparison of serum levels of calcium and magnesium among preeclamptic and normotensive pregnant women at Nnamdi Azikiwe University Teaching Hospital, Nnewi, Nigeria. Int J Res Med Sci. 2014;2(2):404–8.

[38] Richards DGD , Lindow SW, Carrara H et al. Van der Spuy ZM. A comparison of maternal calcium and magnesium levels in pre-eclamptic and normotensive pregnancies: An observational case–control study. BJOG. 2013;121:327–36. 24102858 10.1111/1471-0528.12436

[39] Sidahmed MAE , Abubaker NE, Elfadil GA. Serum totaol calcium, magnesium, sodium and potassium in sudanese women with preeclampsia. Int J Adv Res. 2017;5(2):2061–6.

[40] Ugwuja EI , Famurewa AC, Ikaraoha CI. Comparison of Serum Calcium and Magnesium Between Preeclamptic and Normotensive Pregnant Nigerian Women in Abakaliki, Nigeria. Ann Med Health Sci Res. 2016;6:33–37. 27144074 10.4103/2141-9248.180269 PMC4849113

[41] Wakumilua PN , Nzongola-Nkasu DK, Moyene JPE et al. Seric Calcium and Magnesium in Normal and Pre Eclamptic Pregnant Women: A Case-Control Study in Kinshasa, D R Congo. Open J Obstet Gynecol. 2018;8:408–15.

[42] He L , Lang L, Li Y et al. Comparison of serum zinc, calcium, and magnesium concentrations in women with pregnancy-induced hypertension and healthy pregnant women: A meta-analysis. Hypertens Pregnancy. 2016;35(2):1–8. 26930501 10.3109/10641955.2015.1137584

[43] Makrides M , Crosby DD, Bain E et al. Magnesium supplementation in pregnancy. Cochrane Database Syst Rev. 2014;4:1–54. 10.1002/14651858.CD000937.pub2 PMC6507506 24696187

[44] Bullarbo M , Mattson H, Broman A-K et al. Magnesium supplementation and blood pressure in pregnancy: a double-blind randomized multicenter study. Journal of pregnancy. 2018;4843159:1–10. 10.1155/2018/4843159 PMC5996415 30002931

[45] Easterling T , Hebert M, Bracken H et al. A randomized trial comparing the pharmacology of magnesium sulfate when used to treat severe preeclampsia with serial intravenous boluses versus a continuous intravenous infusion. BMC Pregnancy Childbirth. 2018;18(290):1–10. 29976161 10.1186/s12884-018-1919-6 PMC6034206

[46] Tukur J. The use of magnesium sulphate for the treatment of severe pre-eclampsia and eclampsia. Ann Afr Med. 2009;8(2):76–80. 19805935 10.4103/1596-3519.56232

[47] Gimenez-Mascarell P , Schirrmacher CE, Martinez-Cruz LA et al. Novel aspects of renal magnesium homeostasis. Front Pediatr. 2018;6(00077):1–13. 29686978 10.3389/fped.2018.00077 PMC5900390

[48] Raijmakers MT , Dechend R, Poston L. Oxidative stress and preeclampsia: Rationale for antioxidant clinical trials. Hypertension. 2004;44(4):374–80. 15326082 10.1161/01.HYP.0000141085.98320.01

[49] Kanasaki K , Kumagai A. The impact of micronutrient deficiency on pregnancy complications and development origin of health and disease. J Obstet Gynaecol Res. 2021;47(6):1965–72. 33783077 10.1111/jog.14770

[50] Williams PJ , Pipkin BF. The genetics of pre-eclampsia and other hypertensive disorders of pregnancy. Best Pract Res Clin Obstet Gynaecol. 2011;25(4):405–17. 21429808 10.1016/j.bpobgyn.2011.02.007 PMC3145161

[51] Yogi A , Callera GE, Antunes TT et al. Vascular biology of magnesium and its transporters in hypertension. Magnes Res. 2010;23(4):S207–15. 21199786 10.1684/mrh.2010.0222

[52] Swaminathan R. Magnesium Metabolism and its Disorders. Clin Biochem Rev. 2003;24:47–66. 18568054 PMC1855626

